# Sotatercept Versus Selexipag in Severe Pulmonary Arterial Hypertension: An Indirect Comparison of Efficacy Based on an Artificial-Intelligence Method That Reconstructed Patient-Level Data From Three Randomized Trials

**DOI:** 10.7759/cureus.103912

**Published:** 2026-02-19

**Authors:** Andrea Messori, Roberto Brunoro, Maria Gabriella Paolì, Melania Rivano

**Affiliations:** 1 HTA Unit, Regione Toscana, Firenze, ITA; 2 Department of Pharmaceutical Sciences, University of Milan, Milan, ITA; 3 Department of Hospital Pharmacy, Azienda Ospedaliero Universitaria Policlinico “G. Rodolico–San Marco”, University of Catania, Catania, ITA; 4 Department of Hospital Pharmacy, Binaghi Hospital, Cagliari, ITA

**Keywords:** indirect comparisons, ipdfromk method, kaplan-meier curves, pulmonary arterial hypertension, randomized controlled trials, selexipag, sotatercept

## Abstract

Background: Pulmonary arterial hypertension (PAH) remains a progressive and potentially fatal disease despite currently available treatments. Both sotatercept and selexipag have demonstrated clinical benefits in randomized controlled trials (RCTs); however, no direct head-to-head trial has compared these agents. We therefore conducted an indirect comparison using reconstructed individual patient data.

Methods: We performed a systematic search of PubMed, Scopus, and EMBASE to identify placebo-controlled RCTs evaluating sotatercept or selexipag in PAH. Kaplan-Meier curves from eligible trials were digitized and analysed using the artificial-intelligence (AI) algorithm IPDfromKM to reconstruct individual patient data. Only participants classified as WHO functional class II or III were included. Heterogeneity among pooled placebo arms was assessed, and treatment effects were estimated using Cox regression. Results were reported as hazard ratios (HRs) with 95% confidence intervals (CIs).

Results: Four relevant RCTs were identified (STELLAR, GRIPHON, HYPERION, and ZENITH). STELLAR, GRIPHON, and HYPERION satisfied the inclusion criteria, whereas ZENITH was excluded because it enrolled patients in WHO functional class III or IV. Using IPDfromKM, we reconstructed the six study arms from the three included trials. Based on reconstructed data, placebo arms showed no significant heterogeneity (likelihood ratio = 0.64; p = 0.70). Compared with pooled placebo, sotatercept and selexipag produced HRs of 0.26 (95% CI 0.18-0.38) and 0.57 (95% CI 0.47-0.70), respectively. The main indirect comparison demonstrated a statistically significant benefit for sotatercept over selexipag (HR = 0.45; 95% CI 0.29-0.70; p = 0.00036).

Conclusions: AI-based reconstruction of individual patient data made it possible to compare the efficacy of therapies in the absence of direct head-to-head evidence. These findings suggest that sotatercept may reduce PAH-related events more effectively than selexipag, although the inference is derived from reconstructed and indirectly compared data.

## Introduction

Although pulmonary arterial hypertension (PAH) often presents with non-specific symptoms, its progressive nature can result in serious complications, high morbidity and mortality rates, and an impaired quality of life. Many patients are diagnosed late, presenting with worsened symptoms at the start of treatment [1]. Due to the progressive nature of PAH, management aims to delay disease progression, improve risk status and risk score, minimize PAH-related hospitalizations, and optimize exercise capacity. According to the 2022 European Society of Cardiology - European Respiratory Society Pulmonary Hypertension Guidelines [2], effective management of PAH requires frequent follow-up and reassessment in order to optimize a patient’s treatment regimen based on clinical findings, risk status, risk score, worsening PAH symptoms or medication side effects. Sotatercept [3] and selexipag [4] are two recent innovative treatments indicated for use in patients with PAH. These two agents share the common characteristic of being indicated as add-on therapies; on the other hand, they differ in their route of administration because sotatercept is administered subcutaneously (typically one injection every three weeks), whereas selexipag is administered orally. For this reason, the transition from parenteral or inhaled prostacyclin pathway agents to selexipag has attracted considerable interest from clinicians.

PAH is a progressive disease related to remodeling of small pulmonary arteries, leading to increased pulmonary pressure and right ventricular failure. In Western countries, severe PAH (according to WHO functional class II-III) occurs in about 0.2-0.6 new cases per 100,000 people annually. Current therapies, targeting the nitric oxide, endothelin, and prostacyclin pathways, improve hemodynamics and exercise capacity but have not substantially extended survival. This highlights the need for therapeutic options based on alternative mechanisms of vascular remodelling.

Sotatercept and selexipag have emerged as promising agents supported by recent randomized controlled trials (RCTs) [5,6], but no head-to-head study has directly compared their efficacy. In the absence of such trials, indirect comparisons can help generate comparative evidence by integrating data from separate placebo-controlled studies. Therefore, we used an artificial intelligence (AI) tool (the IPDfromKM method [7,8]) designed to reconstruct individual patient data (IPD) from published Kaplan-Meier curves. The tool extracts information from these curves to create a database representing individual patients, including follow-up time and event status. This dataset of “reconstructed” IPD allows researchers to simulate comparative analyses between treatments even when original patient-level data are unavailable.

Our aim was to perform an indirect comparison of sotatercept vs selexipag using the IPDfromKM method. For this purpose, we first identified the placebo-controlled RCTs evaluating each agent; then, the IPDfromKM method was applied to reconstruct trial populations. Finally, we compared the two treatments based on Kaplan-Meier curves of “reconstructed” patients.

## Materials and methods

Literature search and inclusion criteria

To identify eligible RCTs, we searched PubMed, Scopus, and EMBASE.

The literature search from inception to the present date consisted of the search term “sotatercept OR selexipag” combined on the filter of “randomized controlled trials”.

Studies were included if they met the following criteria: (i) RCTs enrolling patients with PAH (WHO functional class II-III) receiving stable background therapy; (ii) reporting a time-to-event endpoint in which the event was a composite of all-cause death or a PAH-related complication; and (iii) providing a Kaplan-Meier curve comparing an active treatment arm with placebo.

Reconstruction of individual patient data

IPD were reconstructed from published Kaplan-Meier survival curves using the IPDfromKM approach [7,8]. KM plots for treatment and placebo arms were digitized with WebPlotDigitizer (version 4.7; available online at https://apps.automeris.io/wpd/, accessed 18 August 2025; distance: 20; Δx and Δy = 15). The extracted X-Y coordinates, together with the number of randomized patients and the number of events, were then processed using the IPDfromKM package (version 1.2.3.0; last updated 22 March 2022; available at https://www.trialdesign.org/one-page-shell.html#IPDfromKM, accessed 15 January 2026).

This procedure produced reconstructed datasets containing survival times (defined as the time from trial enrollment to the last available follow-up) and outcomes, classified as alive/censored or dead/progressed. Reconstructed IPD were generated for each study arm. To reduce the risk of digitization-related errors, the full workflow (curve digitization and IPD reconstruction) was performed independently by two researchers, and outputs were compared for consistency.

Data analysis and statistical tests

Our analysis tested the hypothesis of a different efficacy between sotatercept and selexipag; the methodological framework consisted of an anchored indirect comparison via pooled placebo arms; efficacy was measured according to the primary endpoints of the two trials, which were very similar.

After constructing the reconstructed datasets, indirect comparisons were performed between each active therapy and placebo, as well as between sotatercept and selexipag, using standard time-to-event methods similar to those applied to datasets derived from real patients. Cox multiple regression models were used to estimate hazard ratios (HRs) with 95% confidence intervals (CIs). HRs were first calculated for each active agent versus the pooled placebo arms, and then for sotatercept versus selexipag. Between-trial heterogeneity was evaluated using the likelihood ratio test and the corresponding p-value.

In addition, considering that medians were not reached in the Kaplan-Meier curves of the two active agents, the values of restricted mean survival time (RMST) at 96 weeks were determined according to Royston and Parmar [9].

## Results

The literature search retrieved four placebo-controlled RCTs of interest: STELLAR [5], GRIPHON [6], HYPERION [10], and ZENITH [11]. As shown in Table 1, the first three trials fulfilled our inclusion criteria, whereas ZENITH was excluded because its population consisted of patients in WHO functional class III or IV.

**Table 1:** Main characteristics of the three included RCTs. PAH, pulmonary arterial hypertension; RCT, randomized controlled trial; T, treatment group; C, control group.

Trial	Inclusion criteria	Event definition	Follow-up length	Comparators	Crude event rates	HR reported in the original trial	HR computed from reconstructed patient data§
STELLAR trial: Hoeper et al. 2023 [5]	Patients with PAH (according to WHO functional class II or III) who were receiving stable background therapy	A composite of death from any cause or a complication related to pulmonary arterial hypertension	75 weeks	Sotatercept vs placebo	T=9/163 C=42/160	HR=0.16 (95%CI, 0.08 to 0.35)	HR=0.2417 (95%CI, 0.1208 to 0.4833)
GRIPHON trial: Sitbon et al. 2015 [6]	Patients with PAH who were receiving no treatment or a stable dose of an endothelin-receptor antagonist, a phosphodiesterase type 5 inhibitor, or both.	The same	156 weeks	Selexipag vs placebo	T=155/582 C=242/574	HR=0.60 (99%CI, 0.46 to 0.78)	HR=0.5965 (95%CI, 0.4843 to 0.7347)
HYPERION trial: McLaughlin et al. 2025 [10]	Patients with functional class II or III PAH who had received the diagnosis less than one year earlier, had an intermediate or high risk of death, and were receiving double or triple background therapy.	The same	156 weeks	Sotatercept vs placebo	T=17/160 C=59/160	HR=0.24 (95%CI, 0.14 to 0.41)	HR=0.2392 (95%CI, 0.1415 to 0.404).3
§ In this estimation, no pooling of control groups was performed; the control arm (regarding both real patients and reconstructed patients) was that reported in the original trial.							

**Figure 1:**
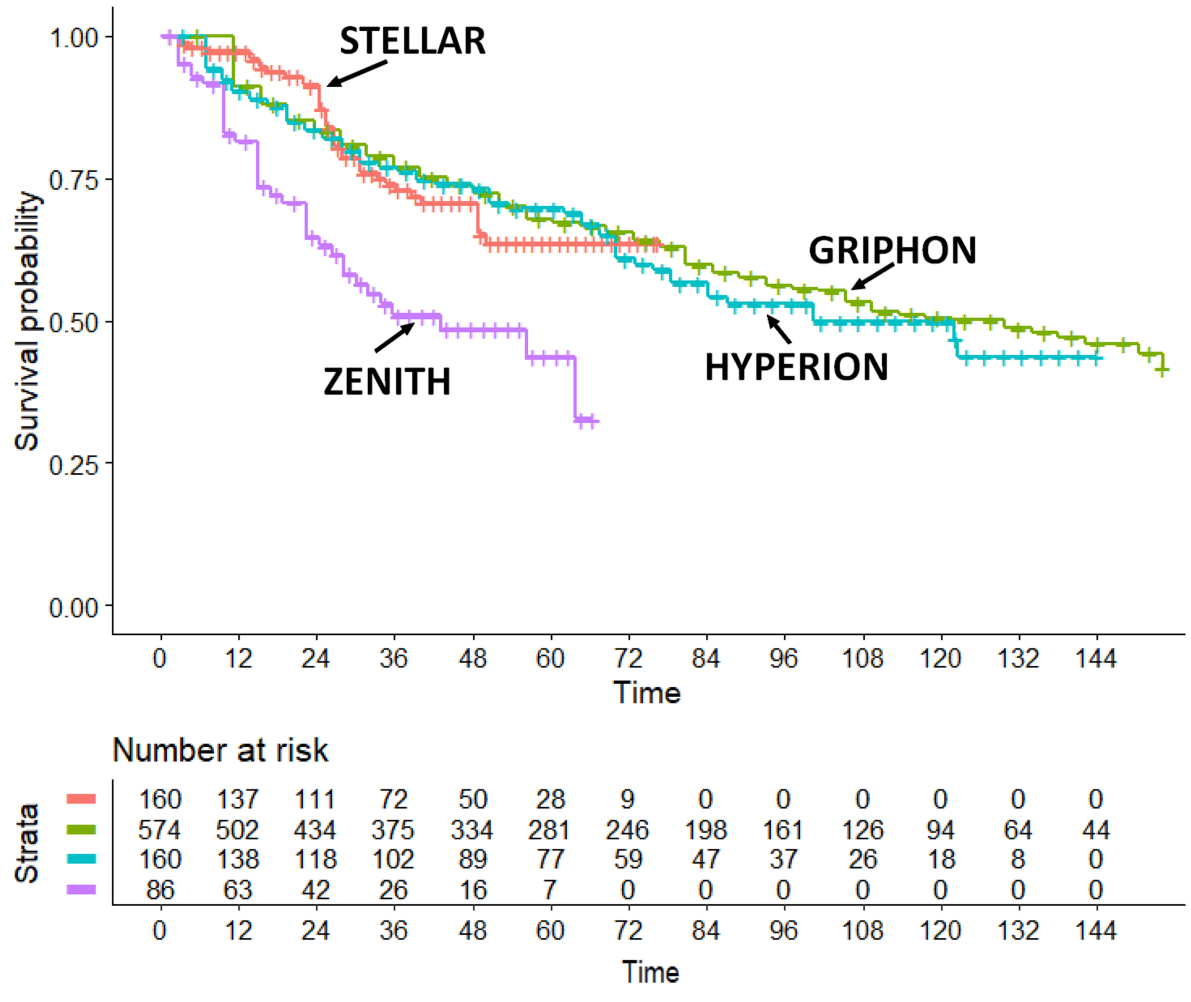
Kaplan-Meier curves generated after reconstruction of individual patient data with assessment of heterogeneity across the four placebo arms. Symbols: STELLAR [5] in red; GRIPHON [6] in green; HYPERION [10] in blue; ZENITH [11] in purple; the panel below the graph shows the distribution of patients at risk over time according to the four trials.

Assessment of heterogeneity indicated that outcomes in the placebo arms of STELLAR, GRIPHON, and HYPERION [5,6,10] were highly consistent (likelihood ratio test = 0.64 on 2 df; p = 0.70). Although similarity in outcomes does not necessarily confirm equivalence of baseline characteristics, the minimal heterogeneity observed across these placebo groups supported the validity of the planned indirect comparison. In contrast, when the placebo arm of ZENITH was included in a separate heterogeneity assessment (Figure 1), heterogeneity increased markedly (likelihood ratio test = 21.9 on 3 df; p < 0.001), consistent with the poorer prognosis of patients enrolled in the ZENITH trial [11]. Finally, our estimation of the difference in the values of RMST at 96 weeks for sotatercept vs selexipag gave the following result: RMST of sotatercept minus selexipag = 7.445 weeks (95%CI, 3.982 to 10.908). 

The Kaplan-Meier curves of the six arms from the three included trials were digitized, and individual patient-level time-to-event data were reconstructed using the IPDfromKM method. This generated reconstructed datasets for each arm, which were then used for pooled placebo analyses and indirect treatment comparisons.

**Figure 2:**
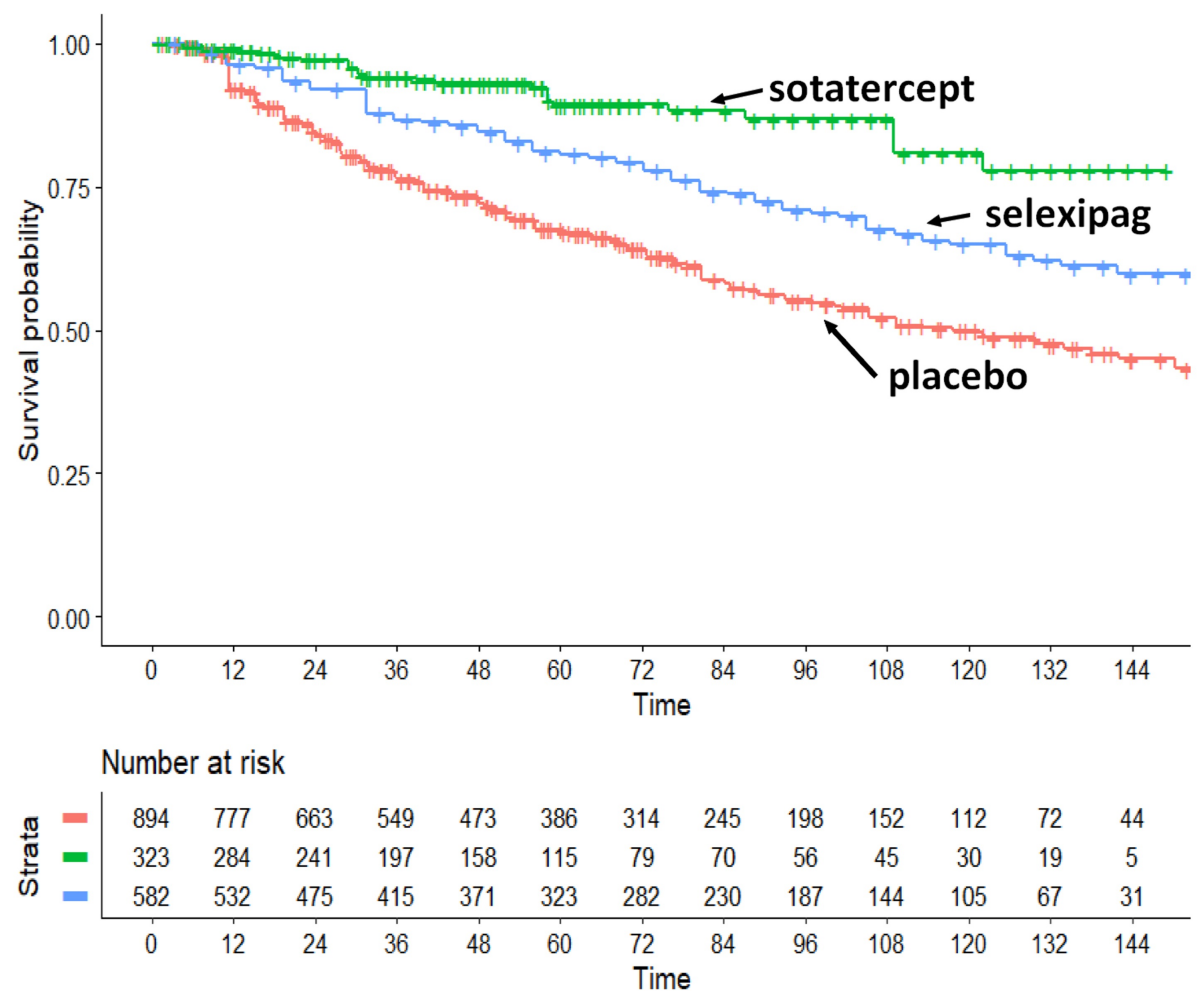
Main analysis: indirect comparison among patients treated with sotatercept or selexipag versus the three placebo arms pooled together. Symbols: sotatercept (in green); selexipag (in blue); three placebo arms pooled together (in red). Endpoint: a composite of death from any cause or a complication related to pulmonary arterial hypertension; time in months; the panel below the graph shows the distribution of patients at risk over time according to the two active treatments and the pooled placebo group.

The primary objective of the study-an indirect comparison between sotatercept and selexipag-showed a statistically significant advantage in favour of sotatercept (Figure 2). When each active therapy was separately compared with the pooled placebo arms, both treatments demonstrated clinically meaningful and statistically significant effects (sotatercept vs placebo: HR = 0.2597; 95% CI 0.1765-0.382; selexipag vs placebo: HR = 0.5742; 95% CI 0.4723-0.698).

Most importantly, the indirect comparison between sotatercept and selexipag suggested a treatment effect favouring sotatercept (HR = 0.452; 95% CI 0.293-0.697; p = 0.00036), representing the key finding of this analysis. Likewise, our estimate of the gain of RMST of 7.445 weeks per patient (over the first 96 weeks of treatment) is helpful to quantify the magnitude of the incremental benefit of sotatercept vs selexipag. It is noteworthy that participants in the HYPERION trial had a more recent diagnosis (<12 months) compared with those enrolled in STELLAR and GRIPHON; however, this difference did not appear to materially influence the overall results. Finally, the last two columns of Table 1 further support the high fidelity of the IPD reconstruction process.

## Discussion

This study illustrates how indirect comparisons based on reconstructed individual patient data can yield clinically relevant insights in settings where RCTs involving real head-to-head patient comparisons are unavailable. Although the inference is derived from reconstructed data and indirect methodology, our findings suggest that sotatercept may offer greater protection than selexipag in patients requiring this therapeutic approach.

Notably, the difference observed between the two agents was highly statistically significant.

The IPDfromKM approach is increasingly used to support indirect survival analyses, particularly in cardiology [12-16] and oncology [17-21]. In the present work, this method allowed pooling of placebo arms across trials, thereby increasing the size and statistical stability of the common comparator group. In addition, it enabled inclusion of a key RCT published only a few months ago [3], which strengthened the relevance of the analysis.

Prior publications have reported that the IPDfromKM method typically provides high-quality reconstructions, with reconstructed survival estimates closely matching those of the original trials [6,7]. In our study, this was supported by the strong agreement between the HR values reported in the final two columns of Table 1.

Among the principal strengths of the IPDfromKM strategy is the retention of event timing, which is often lost in conventional meta-analyses relying on binary endpoints. The method also permits the generation of overlaid Kaplan-Meier curves, which can improve interpretability and facilitate visual comparison.

The limitations of this approach mainly relate to cross-trial heterogeneity, arising from differences in inclusion criteria, background treatments, and follow-up duration among the trials. A further limitation is that IPDfromKM cannot adequately adjust for covariates affecting the time-to-event outcome. Covariate adjustment is only feasible if the original publication provides separate KM curves stratified by the covariate of interest, which is uncommon. Moreover, although real-world data could help complement RCT evidence, integrating real-world datasets with reconstructed patient-level information may be methodologically challenging.

Finally, our article did not focus on the safety of sotatercept and selexipag. Safety aspects of sotatercept and selexipag can be found in the studies by Ahern et al., Chin et al., Bajpai et al., and Elwing et al. [1-4].

## Conclusions

In summary, despite the inherent constraints of indirect comparisons, our results indicate that sotatercept may be more effective than selexipag in this patient population. This study also supports the usefulness of innovative indirect-comparison approaches such as IPDfromKM in generating new evidence for recently developed therapies, particularly when direct head-to-head trials are not available.
