# Correction: Sotatercept Versus Selexipag in Severe Pulmonary Arterial Hypertension: An Indirect Comparison of Efficacy Based on an Artificial-Intelligence Method That Reconstructed Patient-Level Data From Three Randomized Trials

**DOI:** 10.7759/cureus.c411

**Published:** 2026-03-06

**Authors:** Andrea Messori, Roberto Brunoro, Maria Gabriella Paolì, Melania Rivano

**Affiliations:** 1 HTA Unit, Regione Toscana, Firenze, ITA; 2 Department of Pharmaceutical Sciences, University of Milan, Milan, ITA; 3 Department of Hospital Pharmacy, Azienda Ospedaliero Universitaria Policlinico “G. Rodolico–San Marco”, University of Catania, Catania, ITA; 4 Department of Hospital Pharmacy, Binaghi Hospital, Cagliari, ITA

This article has been corrected to fix the following typographical errors:

- Abstract, results paragraph, third sentence: “selexipag and sotatercept” corrected to “sotatercept and selexipag”.
- Results section, fourth paragraph, second sentence: “selexipag vs placebo” corrected to “sotatercept vs placebo”.
- Results section, fourth paragraph, second sentence: “sotatercept vs placebo” corrected to “selexipag vs placebo”.

